# Supplementary material for: Microbial metagenome of urinary tract infection
Source: Sci Rep. 2018 Mar 12;8:4333. doi: 10.1038/s41598-018-22660-8 (PMC5847550; doi:10.1038/s41598-018-22660-8)

**Microbial metagenome of urinary tract infection**

**Supplementary Materials**

Ahmed Moustafa^1*^, Weizhong Li^1,2*^, Harinder Singh^3^, Kelvin J. Moncera^2^, Manolito G. Torralba^2^, Yanbao Yu^3^, Oriol Manuel^4^, William Biggs^1^, J. Craig Venter^1,2,3^, Karen E. Nelson^1,2,3^, Rembert Pieper^1,3#^, Amalio Telenti^2#^

# **Supplementary Figures**

Figure S1. Normalized abundance of 47 bacterial genera across the clinical groups using 16S rDNA.

Figure S2. Normalized abundance of 49 bacterial genera across the clinical groups using whole genome shotgun sequencing.

Figure S3. Correlation between 16S rDNA and whole genome shotgun sequencing.

Figure S4. Differences across sex for selected microorganisms.

# **Supplementary Tables**

Table S1. Clinical data.

Table S2. Clinical data dictionary.

Table S3. 16S sequencing results.

Table S4. Metagenomic sequencing results.

# **Figure S1. Normalized abundance of 48 bacterial genera across the clinical groups using 16S rDNA.** Kruskal-Wallis rank sum test (kruskal.test in R) was performed to compare the number of reads between the three clusters. The *p* value and adjusted *p* value after multiple test correction with Holm–Bonferroni method (p.adjust in R) are shown below the genus name.

# **Figure S2. Normalized abundance of 49 bacterial genera across the clinical groups using whole genome shotgun sequencing.** *P* values, which were calculated as in Figure S1, are shown below the genus name.

**Figure S3. Correlation between 16S rDNA and whole genome shotgun sequencing.** Each dot represents the median normalized abundance for 44 genera identified by the two sequencing approaches. Dots in reds represent those genus that exhibit greater than 2 standard deviation from the regression line, thus suggesting differential performance of one of the two techniques. The lack of correlation between *Raoultella* 16S rDNA and whole genome shotgun sequencing likely result from its recent reassignment as a separate genus from *Klebsiella*.


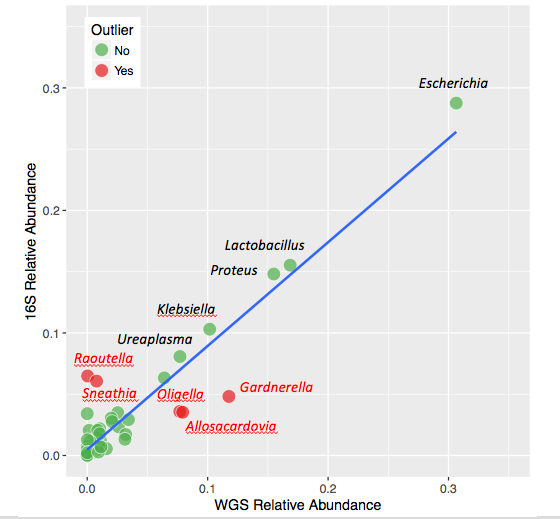


# **Figure S4. Differences across sex for selected microorganisms.** A. Principal component analysis of female and male microbial metagenome showing differences along principal component PC1. B. Boxplot of genera that are nominally statistically different in number of read counts between female and male participants. *P* value (Wilcoxon rank sum test) before and after multiple test correction are shown in B. These differences are not statistically different after multiple testing correction.


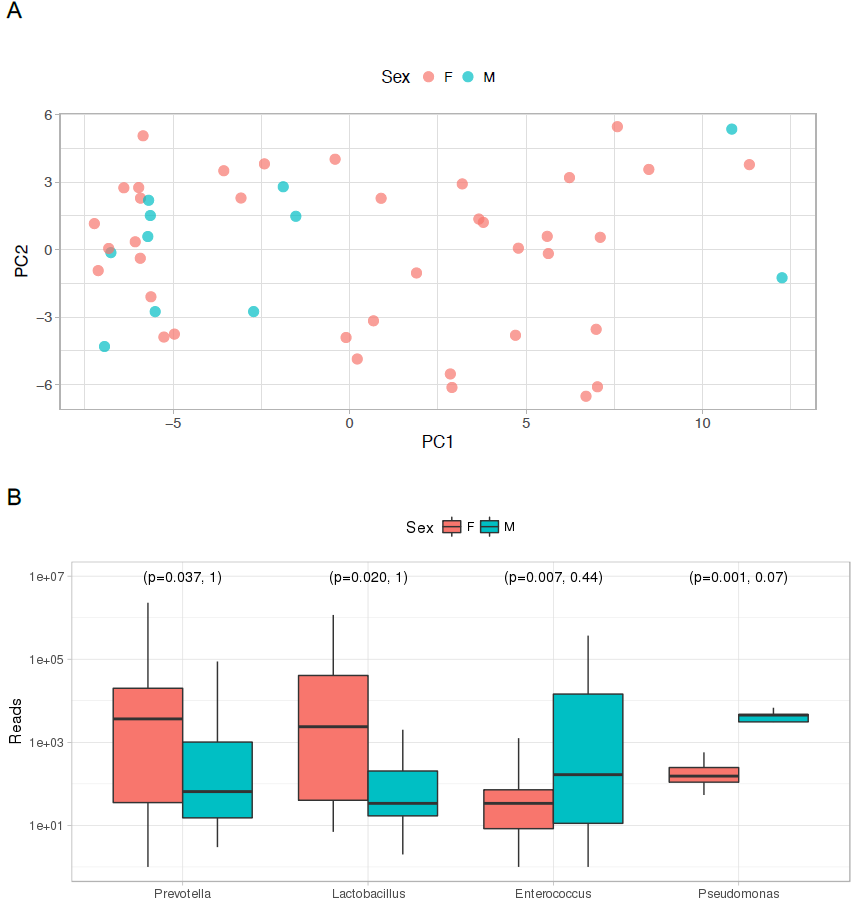

Supplement: Supplementary file 1 — Supplementary materials [file 41598_2018_22660_MOESM1_ESM.docx]
